# Supplementary material for: Evaluation of Antibody Tests for Mycobacterium bovis Infection in Pigs and Deer
Source: Vet Sci. 2023 Jul 27;10(8):489. doi: 10.3390/vetsci10080489 (PMC10458400; doi:10.3390/vetsci10080489)
Supplement: Supplementary file 1 [file vetsci-10-00489-s001.zip › vetsci-2407538-supplementary.pdf]

Barton *et al* - Supplementary data: PARALLEL AND SERIAL COMBINED TESTING

<u>Table S1.</u> Pig Tests - Parallel interpretation for Higher Sensitivity.

	**TB-free**		**TB-positive**	
	**% Specificity (n=402)**		**% Sensitivity (n=29)**	
	**%**	**%**	**%**	**95% CI**
IDEXX/DPP VetTB	**99.0**	98.0-100	**82.8**	69.0-96.5
IDEXX/PPD ELISA	**98.3**	97.0-99.6	**72.4**	56.1-88.7
IDEXX/Enferplex	**98.5**	97.3-99.7	**86.2**	73.6-98.8
DPP VetTB/PPD ELISA	**99.0**	98.0-100	**82.8**	69.0-96.5
Enferplex/DPP VetTB	**99.3**	98.5-100	**93.1**	83.9-100
Enferplex/PPD ELISA	**98.5**	97.3-99.7	**86.2**	73.6-98.8

<u>Table S2.</u> Pig Tests - Serial interpretation for Higher Specificity.

	**TB-free**		**TB-positive**	
	**% Specificity (n=402)**		**% Sensitivity (n=29)**	
	**%**	**95% CI**	**%**	**95% CI**
IDEXX/DPP VetTB	**100**		**72.4**	56.1-88,7
IDEXX/PPD ELISA	**99.8**	99.4-100	**62.1**	44.4-79.8
IDEXX/Enferplex	**99.8**	99.4-100	**72.4**	56.1-88.7
DPP VetTB/PPD ELISA	**100**		**62.1**	44.4-79.8
Enferplex/DPP VetTB	**100**		**75.9**	60.3-91.5
Enferplex/PPD ELISA	**99.8**	99.4-100	**62.1**	44.4-79.8

<u>Table S3.</u> Deer Tests - Parallel interpretation for Higher Sensitivity.

**Parallel – High Se**	**TB-free**		**Farmed TB-positive**		**Park/Wild TB-positive**	
	**% Specificity (n=410)**		**% Sensitivity (n=77)**		**% Sensitivity (n=105)**	
**Combined Tests:**	**%**	**95% CI**	**%**	**95% CI**	**%**	**95% CI**
IDEXX/DPP VetTB	**98.3**	97-99.6	**83.1**	74.7-91.5	**60**	50.6-69.4
IDEXX/PPD ELISA	**98.05**	96.7-99.4	**84.4**	76.3-92.5	**56.2**	46.7-65.7
IDEXX/Enferplex	**98.8**	97.7-99.9	**88.3**	81.1-95.5	**59**	49.6-68.4
DPP VetTB/PPD ELISA	**98.05**	96.7-99.4	**88.3**	81.1-95.5	**60**	50.8-69.4
Enferplex/DPP VetTB	**98.54**	97.4-99.7	**91**	84.6-97.4	**61**	51.7-70.3
Enferplex/PPD ELISA	**98.8**	97.7-99.9	**88.3**	81.1-95.5	**57.1**	47.6-66.7

<u>Table S4.</u> Deer Tests - Serial interpretation for Higher Specificity.

**Serial – High Se**	**TB-free**		**Farmed TB-positive**		**Park/Wild TB-positive**	
	**% Specificity (n=402?)**		**% Sensitivity (n=77)**		**% Sensitivity (n=105)**	
**Combined Tests:**	**%**	**95% CI**	**%**	**95% CI**	**%**	**95% CI**
IDEXX/DPP VetTB	**99.76**	99.3-100	**71.4**	60.9-81.1	**53.3**	43.8-62.8
IDEXX/PPD ELISA	**100**		**74**	64.2-83.8	**50.5**	40.9-60.1
IDEXX/Enferplex	**99.76**	99.3-100	**75.3**	65.7-84.9	**52.4**	42.8-62
DPP VetTB/PPD ELISA	**100**		**68.8**	58.5-79.1	**48.6**	39-58.2
Enferplex/DPP VetTB	**100**		**72.7**	62.7-82.7	**53.3**	43.8-62.8
Enferplex/PPD ELISA	**99.76**	99.3-100	**77.9**	68.6-87.2	**50.5**	40.9-60.1
